# In situ 3D crystallographic characterization of deformation-induced martensitic transformation in a metastable Fe–Cr–Ni austenitic alloy by X-ray microtomography

**DOI:** 10.1038/s41598-024-65505-3

**Published:** 2024-06-24

**Authors:** Osamu Takakuwa, Tatsuya Iwano, Kyosuke Hirayama, Hiroyuki Toda, Akihisa Takeuchi, Masayuki Uesugi

**Affiliations:** 1 Department of Mechanical Engineering, Kyushu University, 744 Motooka, Nishi-ku, Fukuoka, 819-0395 Japan; 2 Graduate School of Mechanical Engineering, Kyushu University, 744 Motooka, Nishi-ku, Fukuoka, 819-0395 Japan; 3 Department of Materials Science, Kyoto University, Yoshida-honmachi, Sakyo-ku, Kyoto, 606-8501 Japan; 4 Japan Synchrotron Radiation Research Institute, 1-1-1 Kouto, Sayo-cho, Sayo-gun, Hyogo, 679-5198 Japan

**Keywords:** Metals and alloys, Characterization and analytical techniques, Imaging techniques

## Abstract

Excellent strength–ductility balance in metastable Fe–Cr–Ni austenitic alloys stems from phase transformation from austenite (fcc structure) to α*ʹ* martensite (bcc structure) during deformation, namely deformation-induced α*ʹ* martensitic transformation (DIMT). Here, DIMT in a metastable Fe–17Cr–7Ni austenitic alloy was detected in situ and characterized in three dimensions (3D) by employing synchrotron radiation X-ray microtomography. This technique utilizes refraction contrast, which is attributable to the presence of phase boundaries between the parent austenite and the newly formed α*ʹ* martensite phase. By combining microtomography and position-sensitive X-ray diffraction, we succeeded in crystallographically identifying multiple α*ʹ* martensite phases continuously transformed in four groups from a single parent austenitic phase.

## Introduction

Metastable Fe–Cr–Ni austenitic alloys display excellent strength–ductility balance because of transformation-induced plasticity (TRIP), which is generated by phase transformation from austenite (fcc structure) to α*ʹ* martensite (bcc structure) during deformation^1,2^, the so-called deformation-induced α*ʹ* martensitic transformation (hereinafter, DIMT). However, when the newly transformed α*ʹ* martensite phase is subjected to a hydrogenated environment, substantial loss of strength and ductility occurs because of damage initiation and evolution^3–5^. Thus, DIMT influences the mechanical properties of metastable Fe–Cr–Ni alloys in both positive and negative ways. There is substantial interest in this technique. Several studies have been performed in which, during DIMT, nuclei form at the intersection of slip bands, ε martensite phases^6,7^, and deformation twins^8,9^. The characteristics of DIMT depend on the stacking fault energy^9^ and the stability of the austenite phase^10^, namely its chemical composition^11^. The newly formed α*ʹ* martensite satisfies a specific crystallographic orientation relationship with the parent austenite (the Kurdjumov–Sachs (K-S) relation^12^) in the form of 24 variants derived from six parallel close-packed directions pertaining to four habit planes. Note that not all the variants are selected in DIMT: some form preferentially over others. In other words, there is variant selection^8,9,13–15^, which depends on the features of the parent austenite grain, such as the shape, crystallographic orientation, and mechanical conditions. Variant selection during the deformation process appears to have substantial effects on the resultant mechanical responses. Even though the phenomenological theory of DIMT, including the theory of its nature and evolution with a focus on variant selection, has been frequently discussed, there is still a lack of linkage between the DIMT process and how it intrinsically influences mechanical properties such as strength, ductility, and fracture toughness. To be able to associate the DIMT process with the resultant mechanical responses, we need to obtain three-dimensional (3D) information on the nature and growth characteristics of the newly formed α*ʹ* martensite, including its structure and shape, as well as its crystallographic orientation in relation to the parent austenite. X-ray^16,17^ and neutron diffraction^18–20^ analyses can be used effectively to acquire hitherto controversial crystallographic information, as well as information on the internal stresses and microscopic strains applied to various diffractive planes during tensile deformation, and hence the dislocation density. Diffraction observations can precisely capture the steep changes in the volume fraction of the α*ʹ* martensite and parent austenite phases as DIMT progresses. However, the information is averaged over the target, making it technically difficult to investigate DIMT in a localized region in 3D.

X-ray computed tomography (CT) is an effective way of collecting internal 3D information^21^. It has been widely applied to lightweight structural materials such as Al^22–24^ and Ti^25–27^ alloys. As X-ray sources become more powerful, its application range has been expanding, even to Fe-based alloys, including multi-phase TRIP (transformation-induced plasticity) steels^28^. However, there is as yet no methodology for detecting the martensite phase that has newly formed and evolved in the parent austenite phase during the deformation of Fe–Cr–Ni austenitic alloys, unlike the case of TRIP in steel, which already has austenite and martensite phases, with a relatively high carbon content, in the initial microstructure. Here, we took particular note of the subtle density differences between the austenite phase (matrix phase: fcc) and the martensite phase (bcc) newly generated by DIMT. The interface of these phases can be detected by enhancing the refraction contrast of projected X-rays; this contrast is derived from density differences.

As a first step, we demonstrated that DIMT of metastable Fe–Cr–Ni austenitic alloys can be continuously detected and characterized in 3D during tensile deformation, and crystallographic information can be obtained by applying pencil-beam X-rays. We succeeded in crystallographically identifying multiple martensitic phases nucleated from a single parent austenitic phase, along with their {111} habit planes.

## Results and discussion

**Figure 1:**
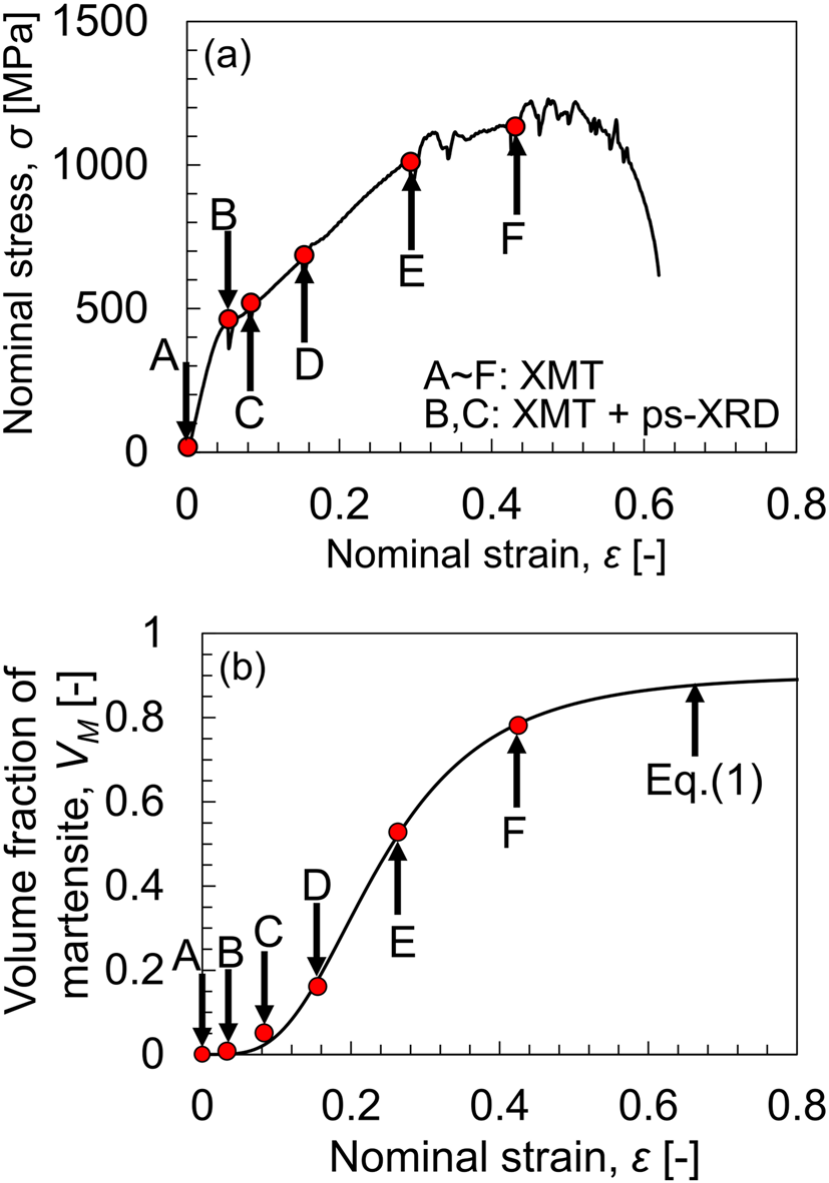
Tensile properties together with DIMT behavior. (**a**) Nominal stress–strain response. (**b**) Increase in volume fraction of the α*ʹ* martensite phase according to Eq. (1), proposed by Olson and Cohen^29^.

We plotted the nominal stress–strain response (Fig. 1a). The nominal strain applied to the specimen was calculated by tracking the displacement of four pairs of pores during the deformation steps. The CT images were collected by X-ray microtomography (XMT) at all deformation steps from A (undeformed) to F, and position-sensitive X-ray diffraction (ps-XRD) was performed at Steps B and C to characterize the parent austenite and α*ʹ* martensite phases. The considerable work hardening seen from Steps B to F was attributed to the hitherto controversial increase in the volume fraction of the α*ʹ* martensite phase, *V*_*M*_, (Fig. 1b), as assessed by XMT (described later). The approximation line is given by Eq. (1), proposed by Olson and Cohen^29^:

1 $$ V_{M} = 1 - {\text{exp}}\left\{ { - \beta \left[ {1 - {\text{exp}}\left( { - \alpha \varepsilon } \right)}\right]^{n}}\right\} $$

where *α* (= 5.99) denotes the rate of shear-band formation and *β* (= 2.30) is proportional to the probability of an intersection of shear bands forming a martensite embryo. *n* is the exponent of 4.91, and *ε* is the nominal strain. The DIMT behavior corresponds closely to sigmoidal variation with respect to the macroscopic strain applied, giving a result similar to that obtained by Talonen and Hänninen for type 301LN stainless steel^30^.

**Figure 2:**
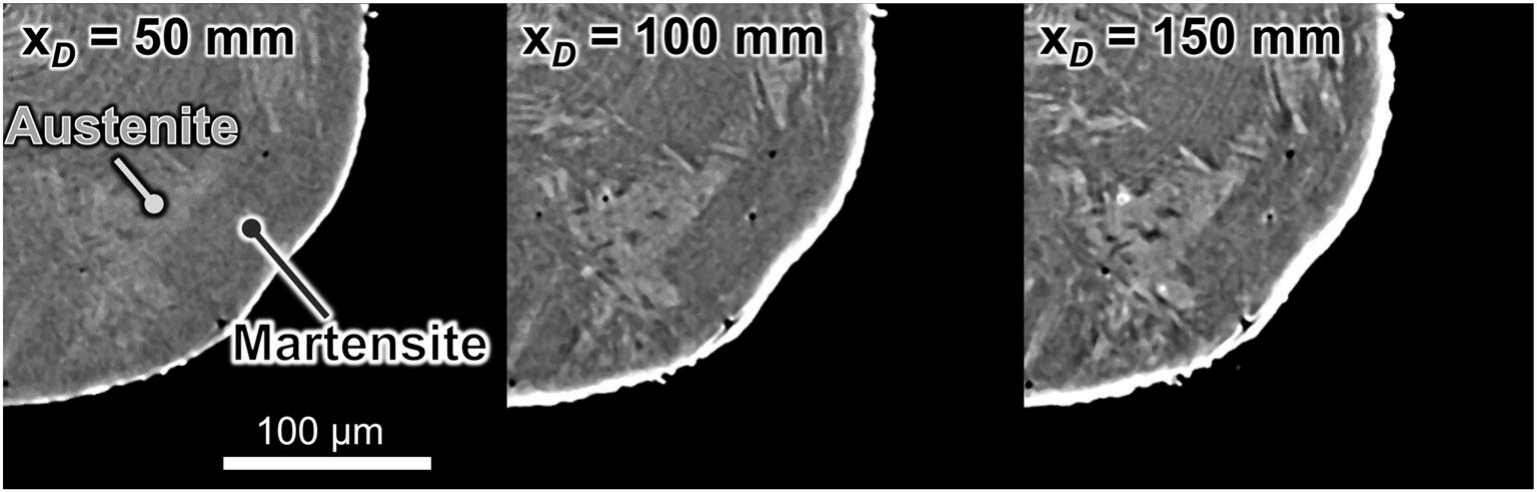
Visualization of parent austenite phase and α*ʹ* martensite phase in tomographic images by enhancing the refraction contrast between them by varying the specimen-to-camera distance.

We obtained tomographic images at a nominal strain, *ε*, of 71% and with specimen-to-camera distances of 50, 100, and 150 mm (Fig. 2). Note that the parent austenite phase and the newly formed martensite phase produced by DIMT are identifiable by XMT. The phase boundary becomes apparent as the specimen-to-camera distance increases owing to enhanced refraction contrast. As the primary objective of this technique is to discriminate the two phases as clearly as possible, we set the distance to 150 mm in subsequent XMT experiments.

**Figure 3:**
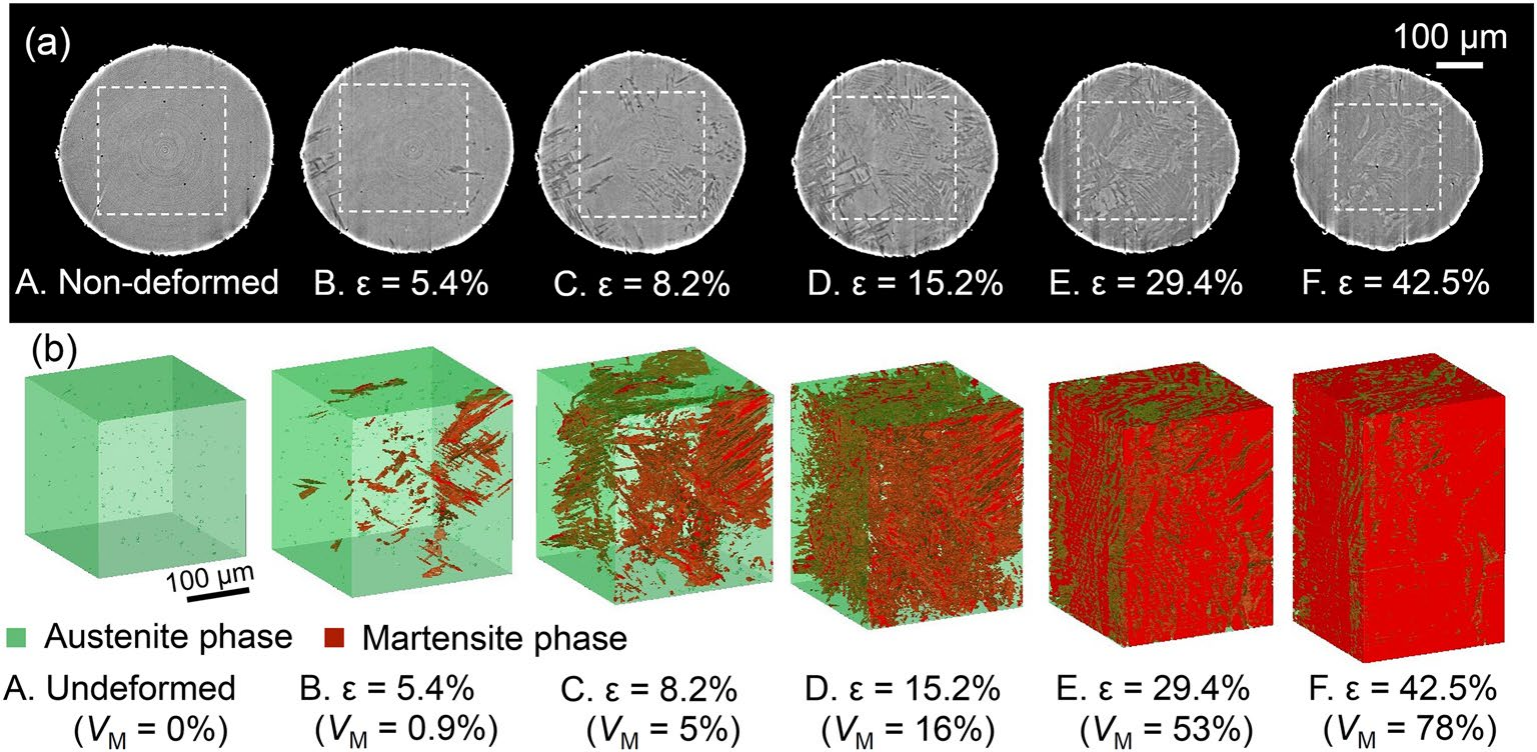
Progress in DIMT through tensile deformation. (**a**) Reconstructed tomographic images; (**b**) 3D images at all deformation steps. In (**b**), the austenite phase is green, and the α*ʹ* martensite phase is red.

**Figure 4:**
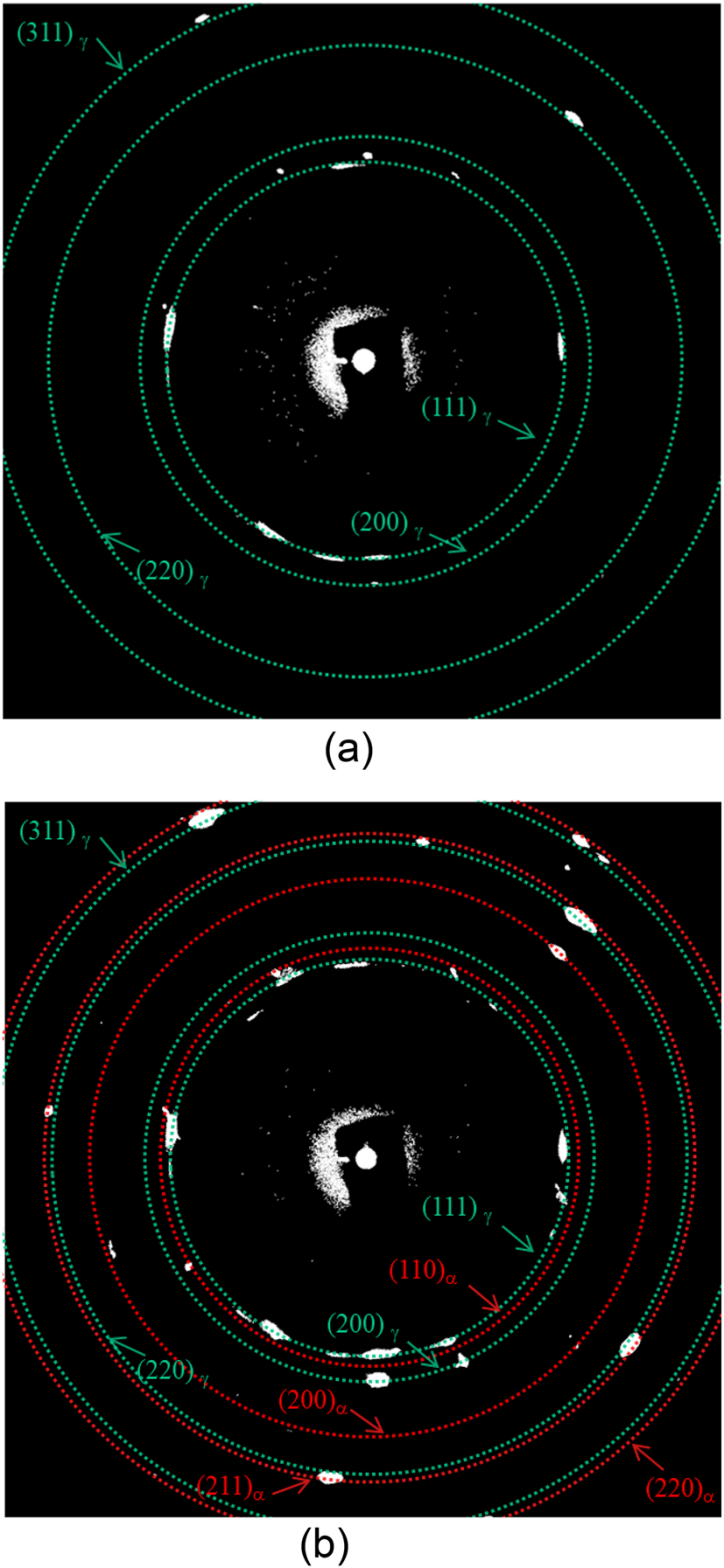
X-ray diffraction spots of the austenite and α*ʹ* martensite phases, acquired by ps-XRD. (**a**) Undeformed. (**b**) Nominal strain of 5.4%. The diffraction rings in green are from the austenite phase, and those in red are from the α*ʹ* martensite phase.

We then obtained tomographic images at each deformation step (cf. A to F in Fig. 1) (Fig. 3a) and 3D images of the austenite phase and the martensite phase formed by DIMT, as identified by using binary coded processing (Fig. 3b). No structures with substantial image contrast are found in the images at Step A in Fig. 1a, suggesting that only the parent austenite phase was present before tensile deformation. At *ε* = 5.4% (Step B), the image contains regions with gray values that are obviously different from the austenite phase and that evolved as the deformation progressed, as manifested in Steps B to F. Moreover, DIMT was detected by X-ray diffraction by comparing before loading and at *ε* = 5.4% (Step B), as shown in Fig. 4, which was acquired by ps-XRD at the same tracked position and rotation angle. The occurrence of the spots from bcc in the red circles is clear evidence that the changes in the gray value stemmed from DIMT. The 3D images (Fig. 3b) indicate that DIMTs are plate-like and develop in clusters of several groups that share the same growth direction, as represented in Step B. We, therefore, demonstrated that the hitherto controversial formation behavior of DIMT can be tracked continuously in 3D by XMT and by enhancing the refraction contrast.

**Figure 5:**
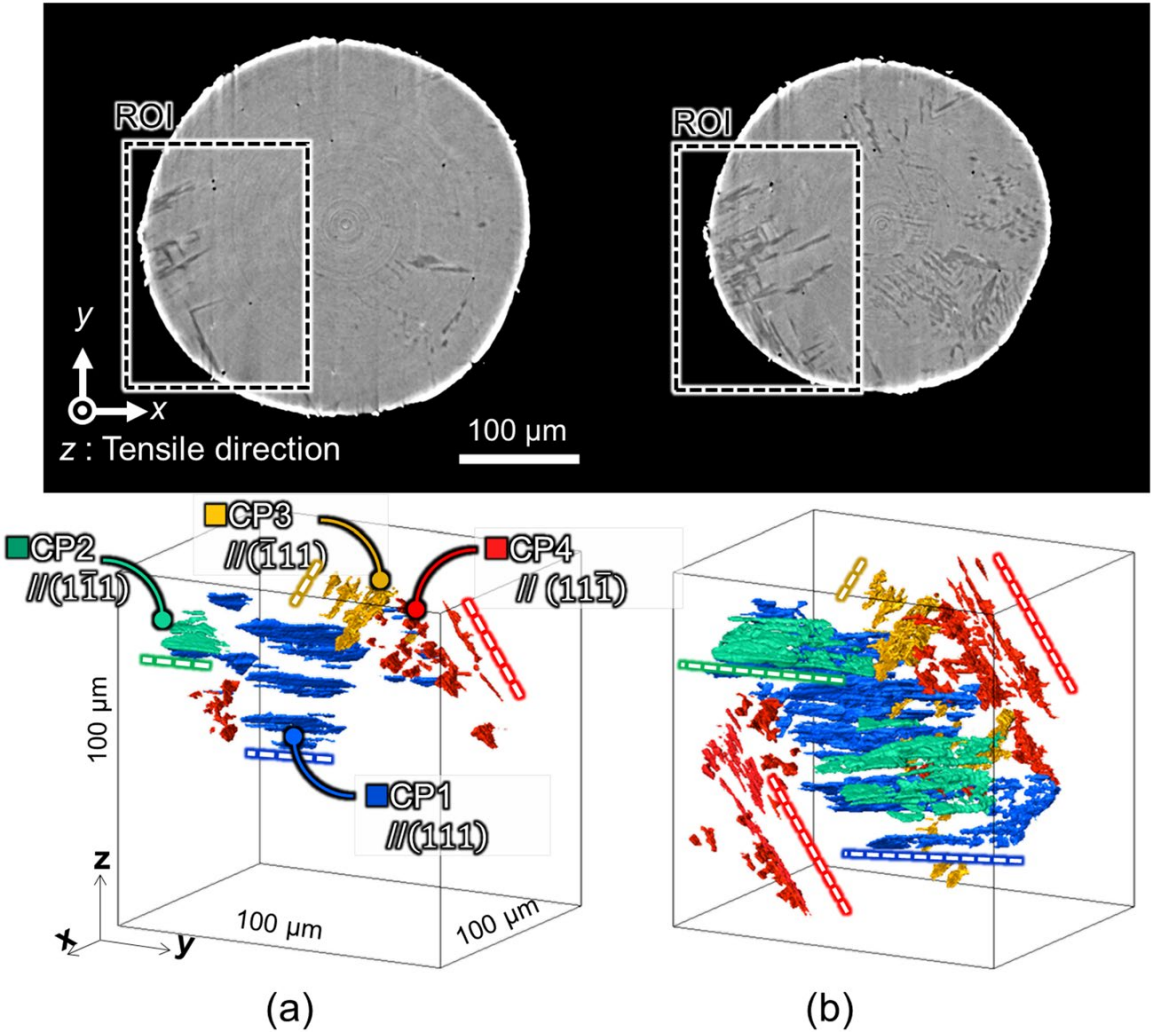
3D crystallographic identification of groups of the martensite phase extracted from the region of interest (dashed boxes) in the tomographic images at *ε* = 5.4% and 8.2%. Groups parallel to (111) in blue, (1–11) in green, (-111) in yellow, and (11–1) in red correspond to CP1, CP2, CP3, and CP4, respectively, in the K-S relationships. (**a**) ε = 5.4%. (**b**) ε = 8.2%.

To characterize each newly formed martensite phase, we obtained 3D views of the phase extracted from the region of interest in the tomographic images at *ε* = 5.4% and 8.2% (Fig. 5). We confirmed by ps-XRD that the martensite phase was newly generated within the same parent austenite grain. By comparing the martensite phases at *ε* = 5.4% and 8.2% in the 3D images, we classified the plate-like martensite phase into four color-coded groups with distinct misorientations. Matching of the diffraction spots from the austenite phase in the immediate vicinity of each martensite group acquired by ps-XRD to their 3D positions by XMT revealed that the martensite phase had formed and developed along four discrete {111} planes in the austenite phase, i.e., the habit planes of the DIMT. This series of results shows that a plate-like martensite phase grew at certain intervals along the {111} habit planes, and that its behavior varied for each close-packed (CP) plane group. The volume fractions, *V*_*M*_, of the martensite phase for each CP group in the K-S relationship (*V*_*M*_ = 34%, 3%, 27%, and 36% for CP1 to C4) were correlated with the maximum Schmid factor, *S*_*F*_, of the {111} < 110 > slip system with respect to the tensile direction (*S*_F_ = 0.45, 0.27, 0.39, and 0.43 for CP1 to CP4). Furthermore, we confirmed the same pattern for the {111} < 112 > slip system, assuming gliding due to Shockley partial dislocation. The α*ʹ* martensite phase formed more abundantly as CP1 and CP4, which had higher Schmid factors, than as CP2, which had a lower one. DIMT occurred preferentially along the {111} habit plane, where the shear stress impinging on the {111} slip plane was higher, and the primary slip system was easier to activate. Investigations have shown the presence of embryos of the α*ʹ* martensite nucleate at the intersection of the shear bands (a comprehensive term for overlapping stacking faults); the embryos grow by repeated nucleation and coalescence^31^. Consequently, leading Shockley partial dislocations gliding on successive {111} planes, or on every second plane, produce deformation twin bundles^32^ and ε martensite phases^6^. If the shear stress on the {111} slip plane is below a certain level, the shear bands are initially parallel. Beyond that level, the number of intersections increases, resulting in extensive nucleation and growth of the α*ʹ* martensite phase^30^. This phenomenon explains the results that we acquired by in situ 3D characterization. Furthermore, when DIMT is viewed in terms of two-dimensional information (e.g., SEM-EBSD; scanning electron microscopy with electron backscatter diffraction), the results will vary depending on which plane is targeted (see Fig. 5). In situ 3D characterization should help to give us a more comprehensive understanding of DIMT.

In summary, DIMT in metastable Fe–Cr–Ni austenitic alloys can be tracked continuously in situ in 3D by using XMT to detect subtle differences in density between the parent austenite and the newly formed martensite phase through X-ray refraction contrast. The result can be characterized crystallographically by combining this technique with ps-XRD. In addition to identifying the habit planes by using the techniques developed here, it is highly possible to analyze misorientations between the parent austenite and the transformed α*ʹ* martensite phase—that is to identify variants in the local region in 3D. We plan to advance this in situ 3D characterization technique further. We hope to achieve a much higher resolution so as to focus on more localized DIMT in the future and to associate the results with several of the mechanical properties that result from DIMT.

## Methods

The material examined was a 40 mm-diameter metastable type 301 austenitic stainless steel, fabricated by hot drawing. The chemical composition of the alloy was Fe–17.3Cr–7.3Ni–0.98Mn–0.069C mass %. Solution annealing was performed at 1423 K for 2 h. The average grain size was about 83 μm. A specimen with a gauge length of 0.7 mm and a cross-sectional area of 0.6 × 0.6 mm was extracted by electrical discharge machining. The gauge section was circularized by mechanical polishing and then electropolished to a diameter of 0.3 mm. Synchrotron radiation X-ray experiments were performed at the BL20XU beamline at SPring-8 at an X-ray energy of 37.7 keV. For projection-type XMT, to enable us to achieve an effective voxel size of 0.5 μm, the setup combined a 4-megapixel CMOS camera (ORCA-Flash 4.0, Hamamatsu Photonics K.K., Hamamatsu, Japan) with a 20 μm-thick GAGG (Gd_3_Al_2_Ga_3_O12: Ce^+^) scintillator. A 3D image was reconstructed from 1800 projection images acquired while the specimen was being rotated through 180° with an exposure time of 150 ms per 0.1°. Diffraction spots in the local regions were acquired while the 3D position was controlled by using the ps-XRD technique with a collimated beam down to 10 × 5 μm (horizontal × vertical direction). The raster scanned every 30 rows × 30 columns for a total of 300 × 150 μm (horizontal × vertical direction). The X-ray energy was 37.7 keV, the same as in XMT. The visible light-conversion-type X-ray camera comprised a CMOS (ORCA-Flash 4.0), optical lenses, and a 20 μm-thick P43 (Gd2O2S: Tb +) scintillator. The sample-to-camera distance was 20 mm. The sample was rotated from 0° to 180° at all scan points during ps-XRD, and diffraction images were acquired every 1° with an exposure time of 80 ms, resulting in 162,000 diffraction patterns. Tensile testing was performed by using a compact material testing machine (CT500, Deben UK Ltd., London, UK) at a displacement rate of 0.2 mm/min.

## Data Availability

The datasets used and/or analyzed during the current study are available from the corresponding author upon reasonable request.
